# A graded prognostic assessment scale to predict overall survival in patients diagnosed with brain metastases undergoing Gamma-knife radiosurgery

**Published:** 2012-11

**Authors:** Sohrab Shahzadi, Parisa Azimi, Mohammad Ali Bitaraf, Maziar Azar, Mazdak Alikhani, Alireza Zali, Sohrab Sadeghi, Soraya Salmanian

**Affiliations:** ^*a*^Department of Neurosurgery, Shahid-Beheshti University of Medical science, Tehran, Iran.; ^*b*^Department of Neurosurgery, Tehran University of Medical science, Tehran, Iran.; ^*c*^Neurosurgeon, Iranian Gamma-Knife Center, Tehran, Iran.; ^*d*^Department of Radiotherapy, Tehran University of Medical science, Tehran, Iran.

## Abstract

**Background:**

The present study aims to evaluate the Graded Prognostic Assessment (GPA) score for predicting overall survival in patients diagnosed with brain metastases undergoing Gamma-knife radiosurgery.

**Methods:**

This was a cross sectional study conducted on the patients diagnosed with brain metastases undergoing Gamma-knife radiosurgery during 2003 to 2011. Clinical and radiological parameters were evaluated, and the GPA score were determined. Kaplan–Meier and log-rank tests were used to assess prognostic factors of the GPA.

**Results:**

Two hundred and twenty patients were eligible to enter the study during the eight years course of study. The mean age of the patients was 54 ± 12.7 years (ranged 19 to 82 years) and were followed up for an average of 7 (range=1-25) months post Gamma-knife surgery. Median survival times according to the GPA were: GPA 0–1, 4 ± 0.4 months; GPA 1.5–2.5, 6 ± 0.7 months; GPA 3, 9 ± 0.9 months; and GPA 3.5–4.0, 12 ± 1.8 months and overall survival were 7 ± 0.6 months. The level of statistical signiﬁcance among GPA groups was p less than 0.0001.

**Conclusions:**

It seems that the preoperative GPA is able to predict Gamma-knife radiosurgery results in patients with brain metastases. However, the results should be confirmed with further clinical trial assessments.

**Keywords:**

Brain metastases, GPA Score, Gamma-knife radiosurgery, Predict


					Volume 4, Suppl. 1 Nov 2012
Publisher: Kermanshah University of Medical Sciences
URL: http://www.jivresearch.org
Copyright:
Congress of Iranian Neurosurgeons, 14-16 November 2012, Kermanshah, Iran
Paper No. 4
A graded prognostic assessment scale to predict overall
survival in patients diagnosed with brain metastases
undergoing Gamma-knife radiosurgery
Sohrab Shahzadi a
, Parisa Azimi a,*
, Mohammad Ali Bitaraf b
, Maziar Azar b
, Mazdak Alikhani c
,
Alireza Zali a
, Sohrab Sadeghi a
, Soraya Salmanian d
a
Department of Neurosurgery, Shahid-Beheshti University of Medical science, Tehran, Iran.
b
Department of Neurosurgery, Tehran University of Medical science, Tehran, Iran.
c
Neurosurgeon, Iranian Gamma-Knife Center, Tehran, Iran.
d
Department of Radiotherapy, Tehran University of Medical science, Tehran, Iran.
Abstract:
Background: The present study aims to evaluate the Graded Prognostic Assessment (GPA) score for predicting
overall survival in patients diagnosed with brain metastases undergoing Gamma-knife radiosurgery.
Methods: This was a cross sectional study conducted on the patients diagnosed with brain metastases undergoing
Gamma-knife radiosurgery during 2003 to 2011. Clinical and radiological parameters were evaluated, and the
GPA score were determined. Kaplan-Meier and log-rank tests were used to assess prognostic factors of the GPA.
Results: Two hundred and twenty patients were eligible to enter the study during the eight years course of study.
The mean age of the patients was 54  12.7 years (ranged 19 to 82 years) and were followed up for an average
of 7 (range=1-25) months post Gamma-knife surgery. Median survival times according to the GPA were: GPA 0-1,
4  0.4 months; GPA 1.5-2.5, 6  0.7 months; GPA 3, 9  0.9 months; and GPA 3.5-4.0, 12  1.8 months and
overall survival were 7  0.6 months. The level of statistical significance among GPA groups was p less than 0.0001.
Conclusion: It seems that the preoperative GPA is able to predict Gamma-knife radiosurgery results in patients with
brain metastases. However, the results should be confirmed with further clinical trial assessments.
Keywords:
Brain metastases, GPA Score, Gamma-knife radiosurgery, Predict
* Corresponding Author at:
Parisa Azimi: Department of Neurosurgery, Imam-Hossain Hospital, Shahid-Beheshti University of Medical Sciences, Imam-Hossain sq., Tehran, Iran.
Email: Parisa.azimi@gmail.com, (Azimi P.).

				

